# Personal

**Published:** 1889-09

**Authors:** 


### Personal.

Dr. William C. Krauss, formerly of Attica, N. Y., but now of this city, was elected a member of the American Society of Microscopists at its recent meeting held here.

Dr. Joseph Price, of Philadelphia, has operated sixteen times for extra-uterine pregnancy, with but one death. The death occurred fifteen days after the operation, and was wholly due to the patient's own indiscretions.

He has also just completed another series of 100 consecutive abdominal sections, without a death, most of which were desperate cases, drainage having been necessary in over fifty per cent. of them.

